# START—the Swiss tele-assisted rehabilitation and training program to support transition from inpatient to outpatient care in the subacute phase after a stroke: feasibility, safety and performance evaluation

**DOI:** 10.3389/fdgth.2024.1496170

**Published:** 2025-01-31

**Authors:** Szabina Gäumann, Carina Ziller, Nele Paulissen, Frank Behrendt, Zorica Suica, Björn Crüts, Luana Gammerschlag, Katrin Parmar, Hans Ulrich Gerth, Leo H. Bonati, Corina Schuster-Amft

**Affiliations:** ^1^ Research Department, Reha Rheinfelden, Rheinfelden, Switzerland; ^2^ Physiotherapy Department, Reha Rheinfelden, Rheinfelden, Switzerland; ^3^ School of Engineering and Computer Science, Bern University of Applied Sciences, Biel, Switzerland; ^4^ Blended Clinic AI GmbH, Nuremberg, Germany; ^5^ Translational Imaging in Neurology (ThINk) Basel, Departments of Head, Spine and Neuromedicine and Biomedical Engineering, University Hospital Basel and University of Basel, Basel, Switzerland; ^6^ Department of Medicine, University Hospital Münster, Münster, Germany; ^7^ Stroke Center and Department of Neurology, University Hospital Basel, Basel, Switzerland; ^8^ Department of Clinical Research, University of Basel, Basel, Switzerland; ^9^ Department for Sport, Exercise and Health, University of Basel, Basel, Switzerland

**Keywords:** telerehabilitation, stroke, training, coaching, monitoring, feasibility, safety, performance

## Abstract

**Introduction:**

Effective rehabilitation is essential to prevent physical and cognitive decline, but many stroke patients face challenges to maintain rehabilitation efforts after hospital discharge. Telerehabilitation, delivered via digital platforms, represents a promising approach for intensive continuation of stroke rehabilitation after discharge. The Swiss tele-assisted rehabilitation and training program (START), delivered through the Blended Clinic mobile application, seeks to support patients to start during inpatient rehabilitation, continue during the transition to the home environment, continue until outpatient rehabilitation starts and beyond. The study aims to evaluate feasibility, safety and performance of the START program on the Blended Clinic platform during inpatient, transition, and outpatient rehabilitation with patients in the early and late subacute phase after a stroke. Furthermore, patients' functional status, mobility and activity level, and health-related quality of life are monitored.

**Methods:**

This single-center feasibility trial with three measurement sessions will include 40 patients, who will be introduced to START during their inpatient rehabilitation. Patients will continue for 12 weeks post-discharge. For the feasibility assessment, process-, training- and mHealth-related parameter will be evaluated, which include recruitment rate, process-evaluation, safety, adherence, drop-out rate, stability and maintenance of the system, usability, quality, satisfaction, user and program experience, and perceived change. Secondary outcomes will focus on motor function, mobility, quality of life, activity level, heart rate, blood pressure, and performance-based measures.

**Discussion:**

The study's strengths include its foundation in previous usability analyses, which informed refinements to the START program. The study's design is based on the ISO 14155 standard, ensuring high standards for medical device research and supporting the future certification of the START program on the Blended Clinic platform. Potential challenges include patient self-reporting via the mobile application and barriers related to technology use among older adults and older mobile devices. Additionally, the availability of coaching is limited to business hours, which may affect adherence. Despite these challenges, the study's findings will provide insights into the feasibility of mobile-based telerehabilitation and guide the design of a future randomized controlled trial.

**Clinical Trial Registration:**

The study is registered with the Swiss National Clinical Trial Portal (SNCTP000005943), EUDAMED (CIV-CH-24-05-046954), and clinicaltrils.gov (NCT06449612).

## 1 Introduction and rationale

Besides other non-communicable diseases, stroke is the largest contributor to the burden of disease worldwide (1). It is the most common cause of acquired disability in adulthood and the second most common cause of dementia (2). One third of people after a stroke remain permanently disabled in their activities of daily living (3). Non-communicable diseases are mainly responsible for the steady increase in health care costs (4). For example, in 2017, the consequential costs of stroke in 32 European countries, including Switzerland, resulted in an economic burden of approximately 65 billion Swiss francs (5). In 2021, the stroke-related costs in 27 European countries amounted 71 billion US dollars, including long-term care costs, productivity losses but excluding unpaid care for stroke patients (6). It is estimated, that the economic burden of stroke in Europe could rise to 86 billion Euros in 2040 if investments in stroke prevention and treatments fail (7). Therefore, to address the health policy challenges, appropriate measures and strategies are being developed and implemented in Switzerland and worldwide. New possibilities are being sought in the context of technological change to support the rehabilitation pathway of patients, e.g., after a stroke.

After a stroke, numerous patients experience an impairment of motor function, which leads to various functional disabilities (8). Due to effective preventive measures, the age-related incidence of new strokes is nowadays declining, but the absolute number of people affected by stroke is simultaneously increasing, with neurological sequelae occurring in 90% of all patients. In Switzerland, the hospitalization rate of all persons after a stroke was 89% (9). An additional risk for patients is the recurrence of a stroke or the development of secondary diseases due to immobilization. Recurrence rates of 11.2% within 12 months (10) and 15% over a follow-up period of 2 years after stroke (11) have been reported.

It is challenging for patients after stroke to maintain motivation to continue and sustain their training at home at the same intensity after an inpatient rehabilitation. Accordingly, new opportunities need to be explored to make training of specific motor functions and physical activity more accessible and acceptable in the outpatient setting, thus fully utilizing the potential to prevent and mitigate further physical and cognitive impairment. An emerging approach is represented by digital applications that are easily and flexibly accessible from home and provide their users a motivational way of exercise programs. This so-called “telerehabilitation” might thus represent a promising approach for the intensive continuation of stroke rehabilitation after an inpatient hospital stay (12). The existing virtual blended care environment Blended Clinic involving a mobile application provides a training platform for patients after a stroke (13). It comprises three main modules: (1) training, (2) coaching and (3) monitoring. These existing modules have been extended with evidence-based recommendations and training programs to be suitable for the early phase of rehabilitation. An exercise program that can now be started during inpatient rehabilitation, facilitating a seamless transition to outpatient treatment.

The START program, which is provided via the mobile application Blended Clinic, is intended to be used for patients after a stroke in the subacute and chronic phase. The Blended Clinic is a virtual blended care environment that was developed by Blended Clinic AI GmbH (Nuremberg, Germany). The platform consists of a web-based portal for users, a mobile application for patients [Blended Clinic, Version 1.0.1. (iOS), 1.3 (Android)], and a database. The START program has been developed for mobile platforms and includes an evidence-based training program, patient education content, and standardised questionnaires making it suitable for the subacute and chronic phases of rehabilitation for patients after a stroke with varying levels of impairment. START is a rehabilitation program that enables patients and therapists to start during the inpatient rehabilitation period, and supports a seamless transition to the family environment, and continuous support from therapists or coaches until outpatient rehabilitation starts and beyond.

### 1.1 Aim and hypothesis

In the proposed study, we primarily aim to evaluate the START program on the Blended Clinic platform for its feasibility, safety and performance in both inpatient rehabilitation environment and outpatient setting, focusing on patients in the subacute phase after a stroke as primary end-users. We hypothesize that the mobile application-based START program is feasible for patients after a stroke (PaS) in the subacute phase in the inpatient setting and during transition to an outpatient setting. Secondly, we aim to assess descriptive parameters, functional status, mobility and activity level, and health-related quality of life (QoL) in patients after a stroke using the START program.

## 2 Methods

### 2.1 Study design, setting, and registration

Our clinical study is designed as a single-centered observational descriptive feasibility trial focusing on the user experience, safety and the performance of the system. Our study will be conducted at the Reha Rheinfelden in compliance with the Declaration of Helsinki, the ISO14155 standard, the guidelines of the International Council for Harmonisation for good clinical practice as well as national legal and regulatory requirements.

The study received approval from the independent ethics committee of Northwestern and Central Switzerland (EKNZ, reference number: 2024-D0028, BASEC Project-ID 2024-D0028) and the competent authority Swissmedic (reference number: 10001354). The study is registered with the Swiss National Clinical Trial Portal (SNCTP000005943), EUDAMED (CIV-CH-24-05-046954), and clinicaltrials.gov (NCT06449612).

### 2.2 Patients and recruitment

Patients' eligibility criteria are listed in Table 1 below.

**Table 1:** Patients' (primary end-users) inclusion and exclusion criteria.

Inclusion criteria	Exclusion criteria
• Age ≥18 years • Clinically confirmed stroke • Modified Rankin Scale score 1–5 • Able, to read and understand German • Owns a smartphone or a tablet operating on Android version 11/iOS version 14 or higher	• Severe aphasia • Clinically significant concomitant disease states: visual, neurological, cardiorespiratory, psychiatric, or orthopedic limitations that prevent a participant from following the investigator's instructions or limit performance in an exercise program • Recent events such as surgery or other surgical procedures, fractures, which limit the performance in an exercise program • Known or suspected non-compliance, drug or alcohol abuse • Participation in another investigation (with interventional therapy, investigational drug or another medical device) within the 30 days preceding and during the present investigation

For recruitment, we use pragmatic consecutive sampling. During the recruitment period, every inpatient after stroke, who fulfils our eligibility criteria, has the opportunity to participate in the study. Patients will be informed about the study in oral and written form. After a minimum of 24 h, patients will be asked to provide written informed consent for the enrollment in the study. Data collection will start, and three sessions will be scheduled over a period of two to three weeks during inpatient stay. The recruitment period will run from July 2024 to 31. March 2025. No compensation for study participation will be provided.

Study participation will be immediately terminated if one of the following events occurs:

• The patient withdraws written consent.
• The patient decides to withdraw from the study.
• The project team members may exclude a patient from the investigation if any physical or mental condition is present that prevents the patient from participating in the study.

Withdrawal of full consent means that the patient does not wish or is unable to participate further in the feasibility study. Data collected up to withdrawal of consent will be used for analysis.

### 2.3 The virtual blended care environment and the START program intervention

**Figure 1:**
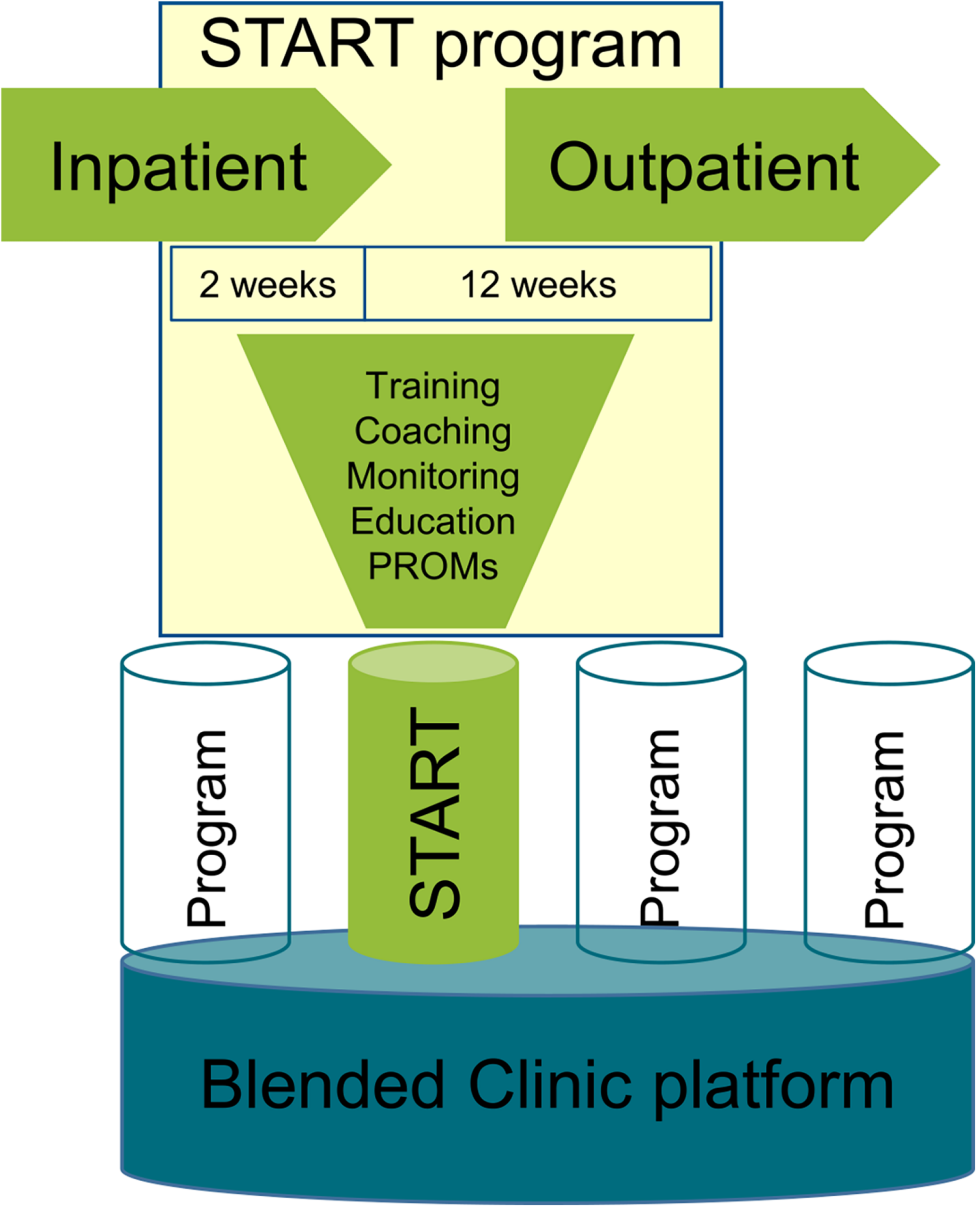
Illustration of the START program and the blended clinic mobile application.

**Figure 2:**
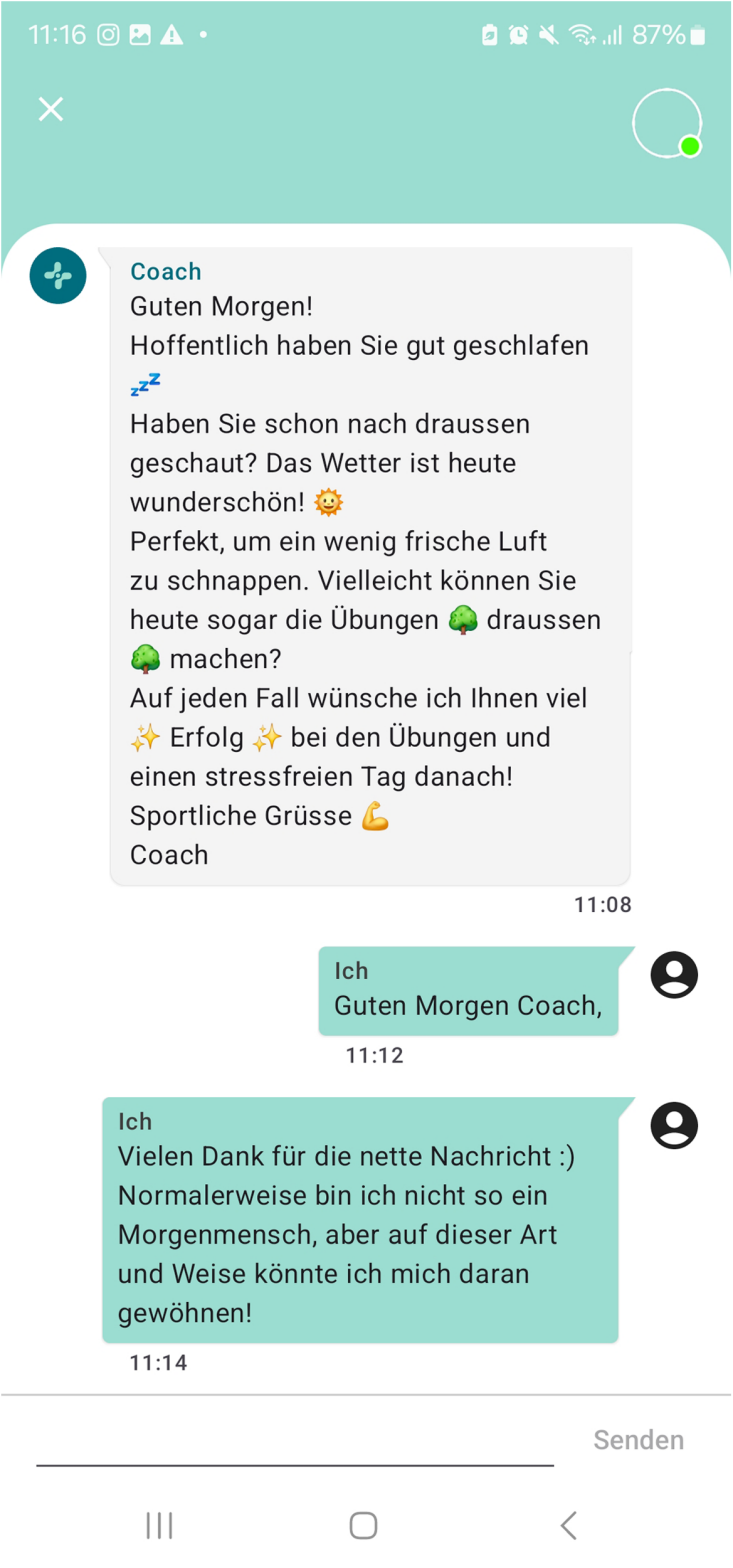
Screenshot of a chat example between patient and coach (German).

**Figure 3:**
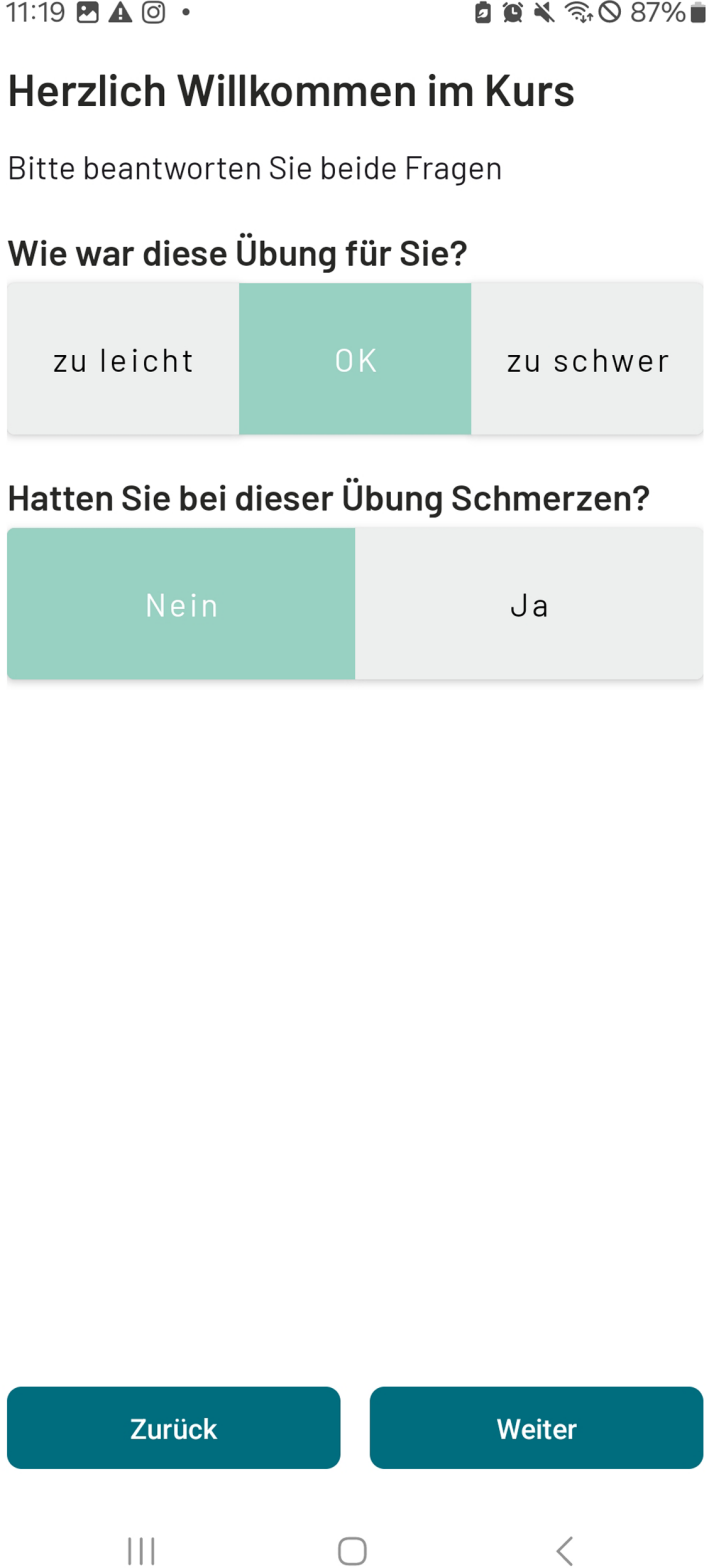
Screenshot of the evaluation options after every exercise (German).

**Figure 4:**
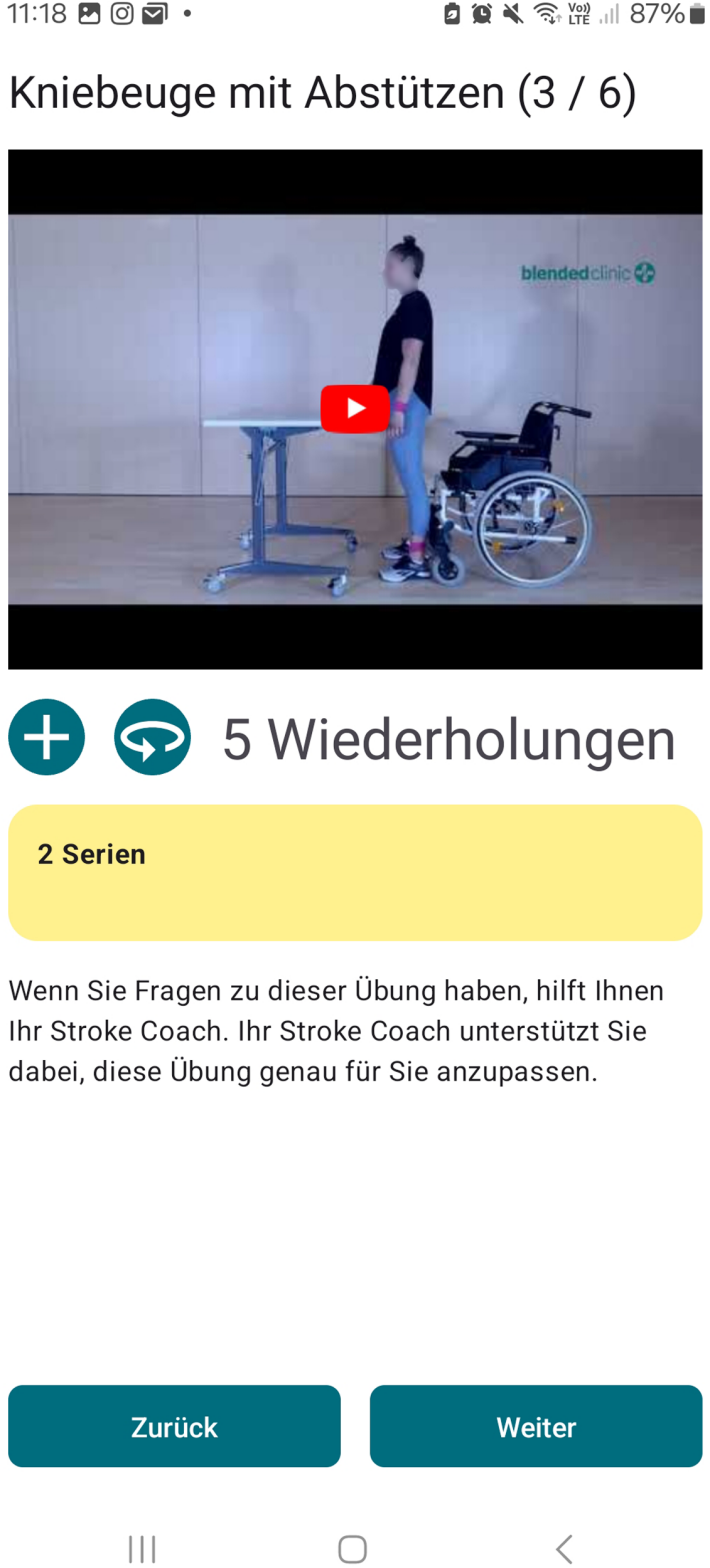
Screenshot of an exercise video example (German). Reprinted with permission from Blended Clinic AI GmbH by Björn Crüts, R&D.

**Figure 5:**
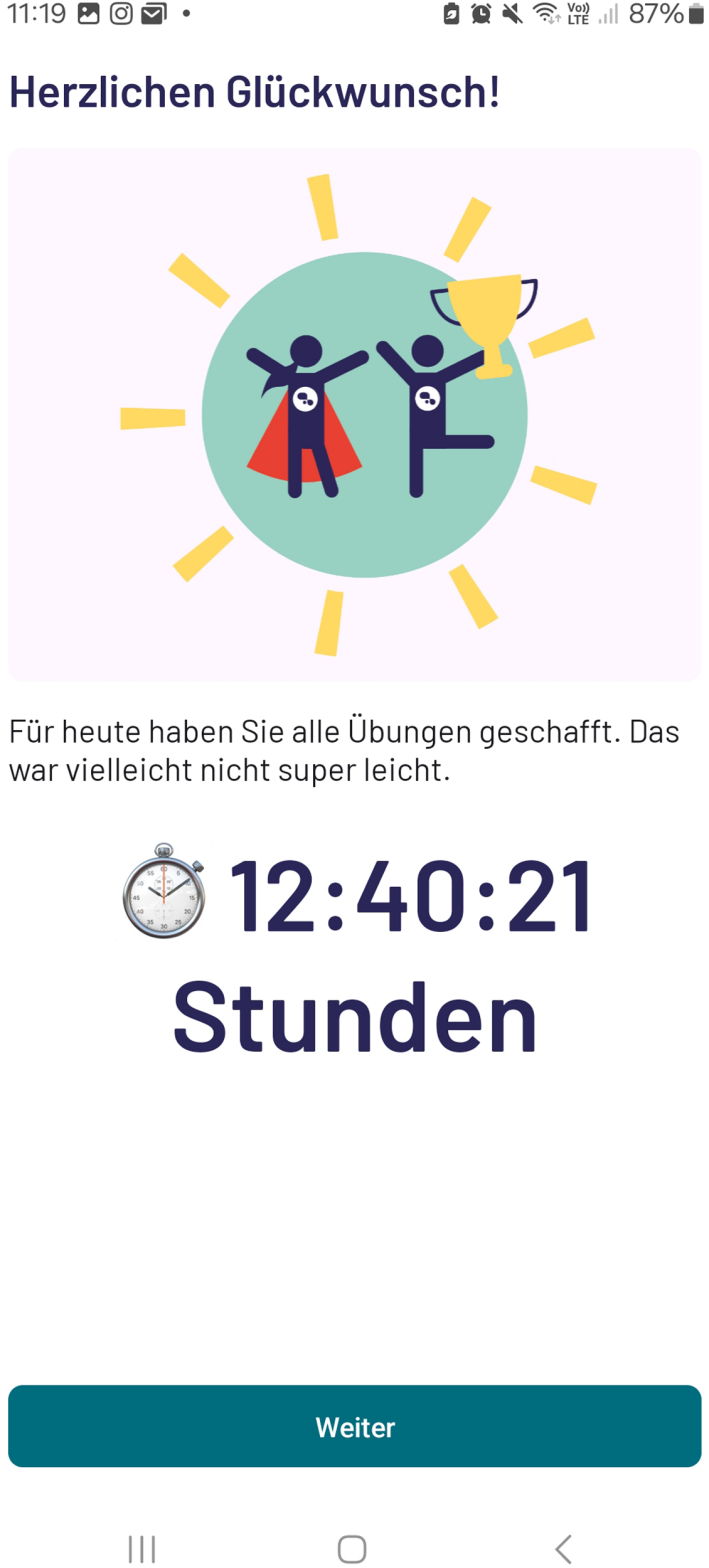
Screenshot of an exercise achievement (German).

The virtual blended care environment Blended Clinic (Nuremberg, Germany) consists of a web-based portal for users, a mobile application for patients [Blended Clinic, Version 1.0.1. (iOS), 1.3 (Android)], and a database. Among others, it contains the START program including: (1) training, (2) coaching, (3) monitoring, (4) patient education and information and (5) patient-reported outcome measures (PROMs). Figures 1–5 provide illustrations of the START program and the Blended Clinic mobile application.

(1) Training: Via the application, patients are provided with a training plan adapted to their individual impairment and rehabilitation goals including exercise videos and description. Progress can be monitored by the coach and the training content can be adapted to the functional level achieved. Every exercise can be rated regarding pain and the patients' perceived effort level: too easy, okay, too hard. Here, the therapist or coach can modify the exercise, its frequency or duration. For different impairment levels, the START program contains seven 3-week familiarization exercise programs intended to be conducted during the inpatient period, and 14 6-week exercise programs for the transition and outpatient period that can be combined among each other depending on patient's progress.
(2) Coaching: Via the integrated chat function in the application, a personal therapist or coach accompanies the patients' individual training, so that motivation is maintained at the highest possible level. Patients receive training advice and motivational message. Questions regarding training and or stroke in general can be clarified promptly with a therapist, who can be reached during business hours on weekdays.
(3) Monitoring: Key parameters of the patient's health status can be entered, displayed and stored in the application, including self-reported blood pressure measurements.
(4) Patient education and information: To inform and guide the patient during their rehabilitation process, podcasts and information messages will be provided. So far, seven podcasts lasting between three and four minute are available focusing on: psychological well-being, sleep, fatigue, nutrition, life style factors, and training.
(5) Patient-reported outcome measures (PROMs) can be send to the patients on pre-defined time points: before, in the middle and after the training period.

Patients will be instructed on how to use and interact with the mobile application within two to three supervised sessions. A trained therapist or coach will introduce patients to the program and application. Patients will be instructed on how to communicate with the personal coach, perform exercises of the individualized training program, how to measure blood-pressure, and enter these date into the application.

The platform offers therapists a web-based dashboard where they can add or create new patient profiles, compile training programs, interact with patients, and control parameters of the monitoring devices. During the therapist training provided by the manufacturer, they will take part in a three day-block course on how to use the web-based dashboard to compile training programs from the exercise pool, to interact with patients, and to control the parameters of the health state. The START program exercise pool contains ready for use training programs tailored to the levels of the modified Rankin Scale. Based on the patients' goal or feedback from the training, the individual selected training program can be modified and adapted to suit the patients' limitations.

#### 2.3.1 START program during inpatient rehabilitation

Besides their multi-disciplinary therapy schedule, patients will receive two to three practice sessions with a therapist lasting 30 to 60 min during three to two weeks before discharge. The intended activities during the sessions are described in Table 2.

**Table 2:** Overview on the practice sessions to get to know the blended clinic application.

Practice session 1	• Onboarding: Under supervision, patients download and set up the mobile application Blended Clinic with the START program on their own smartphone or tablet. They will receive a written short guide for the application including user-friendly explanations with large font size and pictures. • Under supervision, patients learn how to use the application: ○ open, close and navigate through the application menus, ○ read and enter messages for the communication with the personal coach, ○ perform the self-measurement of the blood pressure values and enter the data in the mobile application • Under supervision, patients perform one to eight exercises of their individualized exercise program that was selected by the therapist or coach based on the patient's goal. • Study personnel sets up the activity tracker.
Practice session 2	• Under supervision, patients practice to interact with and navigate through the application menus (open training program, write a message to the coach, ask questions, and enter measured blood pressure data). • Under supervision, patients perform one to eight exercises of their individualized exercise program.
Practice session 3 (optional)	• Under supervision, depending on the patients’ needs, they practice to interact with and navigate through the application menus (open exercise program, write a message to the coach, ask questions, and enter measured blood pressure data). • Under supervision, patients perform one to eight exercises of their individualized exercise program.

Practice sessions one to three will take place in the therapy facilities or the patient's room in the rehabilitation center. Between training sessions with personal contact, patients practice on independently, wear an activity tracker and measure blood pressure daily. Further, they receive coaching messages as well as questionnaires to complete, and they can contact their personal coach during regular working hours.

#### 2.3.2 START program after discharge

After discharge from the inpatient rehabilitation center, patients continue the START program for 12 weeks at home. Patients exercise on their own and measure their blood pressure. They can contact their individual coach via the mobile application during business hours on weekdays. Patients will receive one to five coaching messages per week. For self-evaluation after the sixth week of training, they will complete the modified Rankin Scale, the Stroke Impact Scale, the general health survey SF-36, and the Life Space Assessment via the mobile application.

Their individual coach continuously tracks the patient's progress via the dashboard. After three consecutive days without the patient's adherence to the protocol, e.g., no recorded exercises, or exercising with a total duration of less than 5 min, no blood pressure measurements entries, no messages the therapist will contact the patient via the messenger function in the mobile application. The coach will ask about the patient's well-being and the reasons for non-adherence.

### 2.4 Measurement sessions, outcomes and outcome measures

Patients will be evaluated during three measurement sessions: before the begin of the START program (T1), at discharge (T4), and after completion of the 12-week program (T6). The 12-week program was implemented for two reasons. Firstly, the greatest functional improvements following a stroke are typically observed during the early subacute phase, particularly within the first three months. Secondly, this period coincides with the transition from inpatient to outpatient care and rehabilitation, which we aimed to support and evaluate. All visits will take place in the rehabilitation center. If it is the patient's wish, the last measurement session can take place at the patients' home. Table 3 provides an overview on all measurement sessions, outcomes and outcome measures.

**Table 3:** Summary of scheduled visits, relevant procedures and duration for patients.

Investigation period	Place of visit	Event	Time point	Content	Duration
Enrolment	Inpatient rehabilitation	Patient information	Day −10 to 0	Study information	10 min
		T0	Day 0	Sign informed consent	5 min
		T1	Day 1–7	Baseline assessment: MoCA, mRS, EQ-5D-5l, SPPB, TUG, TIS	20–30 min
Interventional Phase		T2	Week 1 +/− 7 days	Practice session 1 • Introduction to the application Blended Clinic and the START program • Practice to interact with and navigate through the application menus under supervision • Perform 1 to 8 exercises of training program under supervision • Introduction to self-measurement and entering blood pressure values • Set up activity tracker	30–60 min
		Practice	4–14 days	• Patients practice exercises on their own • Patients wear activity tracker and measure blood pressure • Patients receive about 1 to 5 coaching messages • Patients fill in SIS, SF- 36, mRS	5–10 min per training
		T3	Week 2 +/− 7 days	Practice session 2 • Practice to interact with and navigate through the application menus under supervision • Perform 1 to 8 exercises of training program under supervision	30–60 min
		Optional T3b	Week 3 +/− 7 days	Practice session 3 (optional) • Practice to interact with and navigate through the application menus under supervision • Perform 1 to 8 exercises of training program under supervision	30–60 min
		T4	Discharge from inpatient centre	Patients fill in SIS, SF-36, mRS, LSA-G	30 min
	Outpatient rehabilitation	Practice	Week 4–9 +/− 7 days	Patients practice the exercises on their own Patients measure blood pressure	5–10 min per training
		T5	Week 10 +/− 7 days	Patients fill in SIS, SF-36, LSA-G, mRS	30 min
		Practice	Week 11–16 +/− 7 days	Patients practice the exercises on their own Patients measure blood pressure Patients wear activity tracker in the last three weeks of the training program	5–10 min per training
End of Intervention		T6	3 months after discharge +/− 7 days	Patients fill in SIS, SF-36, LSA-G, mRS, SUS, G-MAUQ, AQUA, NPS, Satisfaction (VAS), Perceived change (VAS), FIM, FAC, SPPB, TUG, TIS Feedback Questionnaire, EQ-5D-5l	60–120 min

Comment: mRS, SIS, SF-36 and LSA-G to be filled in via the mobile application.

AQUA, Quality Assessment of Health-Related Apps; FAC, Functional Ambulation Category; FIM, Functional Independence Measure; G- MAUQ, German version of mHealth App Usability Questionnaire; LSA-G, German Life Space Assessment; NIHSS, National Institute of Health Stoke Scale; NPS, Net Promotor Scale; mRS, modified Ranking Scale; SF-36, 36-Item Short Form Healthy Survey; SIS, Stroke Impact Scale; SPPB, Short Physical Performance Battery; SUS, System Usability Scale; TIS, Trunk Impairment Scale; TUG, Timed up and go.

#### 2.4.1 Primary outcome

The primary outcome will be the **feasibility** of the START program on the Blended Clinic platform. Feasibility will be evaluated in three main domains: (1) process-related parameters, (2) training-related parameters, (3) mHealth-related parameters (Table 3).

##### 2.4.1.1 Process-related parameters

• **Recruitment rate:** The number of screened patients vs. included patients will be described including two ratios. First, every patient after a stroke admitted to inpatient rehabilitation vs. excluded patients after the pre-screening of their medical history. Second, potential patients who, received oral and written patient information vs. enrolled participants. Recruitment rate will be assessed throughout the entire study period.
• **Process-evaluation:** The total duration of study-related tasks performed by the study personnel/coach in minutes during the inpatient and the outpatient period will be recorded. This includes the duration of inpatient sessions, coaching (sending questionnaires, answering patient messages, sending educational content, adjusting or setting up new exercise plans), and monitoring health-related parameters.

##### 2.4.1.2 Training-related parameters

• **Safety:** (Serious) adverse events and device deficiencies will be recorded.
• **Acceptance:** Patients satisfaction will be measured with the Net Promoter Score (NPS) at the end of the intervention. The NPS is widely used for measuring user satisfaction. It involves a single question (“How likely is it that you would recommend START to a friend or colleague?”) with an 11-point rating scale (14).
• **Adherence:** The number of completed exercise sessions vs. planned exercise sessions, the duration of completed exercise sessions, the number of completed blood pressure measurements vs. planned measurements and the actual wearing time of the activity tracker vs. the planned wearing time during the pre-defined time period will be recorded.
• **Drop-out rate:** The number and reasons for drop-out will be listed and assessed during the entire investigation period. If possible, the reason for withdrawal will be documented in the eCRF.

##### 2.4.1.3 mHealth-related parameters

• **Stability and maintenance:** The number and description of system errors will be recorded.
• **Usability:** Usability will be measured with the System Usability Scale (SUS). The SUS provides a simple, fast, and reliable tool to assess usability (15, 16). It comprises 10 statements considering the system evaluated on a five-point rating scale from strongly agree to strongly disagree. The SUS was originally developed by Brooke. It allows to evaluate various systems, including hardware, software, mobile devices, applications, etc (17). In addition, patients will fill in the German version of mHealth App Usability Questionnaire (G-MAUQ) (18). The G-MAUQ is designed to assess usability of mHealth apps and consists of 18 items within three subscales: ease of use, interface and satisfaction and usefulness. Patients can rate each item using a 7-point rating scale (from 1 = disagree to 7 = agree).
• **Quality:** The user version of the Quality Assessment of Health-Related Apps (AQUA) (19) consists of 31 items and assesses seven basic dimension of app-quality: usability (4 items), user engagement (5 items), content (4 items), visual design (4 items), therapeutic quality (5 items), impact (4 items), and information (5 items). All items use a five-point rating scale (from 5 = strongly agree to 1 = strongly disagree). For each domain, a sub-score and an overall total score including all domain sub-scores can be calculated.
• **Satisfaction:** Patient's subjective satisfaction will be assessed using a 10 mm-visual analogue scale (VAS) from not satisfied at all to extremely satisfied: “How satisfied are you with the application?”.
• **User and program experience:** will be evaluated using a customised feedback questionnaire with a five-point rating scale (1 = extremely negative to 5 = extremely positive) including specific questions related to the onboarding procedure, program content, coaching and monitoring. It is aimed to gain further insight of participants' opinion towards the START program. The questionnaire addresses joy, design, understanding, functioning and emotions. Additionally, interaction with the devices (blood pressure measurement device, activity tracker, smartphone or tablet) will be evaluated on a five point-rating scale (from 1 = extremely negative to 5 = extremely positive). Furthermore, patients' experience with the coach will be evaluated (how friendly, competent) using a five-point rating scale (1 = extremely negative to 5 = extremely positive).
• **Perceived change:** Patients' subjective global perceived change will be assessed using a visual analogue scale (VAS) from very much worse to very much improved: “How would you rate the change?”.

#### 2.4.2 The secondary outcomes comprise the following

• The Functional Independence Measure (FIM) to evaluate the functional status of patients after a stroke throughout the rehabilitation process. It consists of 18 items focusing on motor and cognitive function. Each category is rated on a 7-point rating scale (from 1 = <25% independence to 7 = 100% independence) (20, 21). The FIM score will be used as a descriptive parameter and is used as a standard evaluation at entry to and discharge from the inpatient period.
• The Functional Ambulation Categories (FAC) rates ambulation ability, determining how much support a patient requires for walking on a six-point rating scale from zero = unable to walk or assistance by walking through two or more assistants to 5 = able to walk independently everywhere (22).
• The Montreal Cognitive Assessment (MoCA) is a screening tool to evaluate mild cognitive dysfunction in different cognitive domains from zero to 30 (no cognitive dysfunction) (23).
• The National Institute of Health Stroke Scale (NIHSS) is a widely used tool by health care providers to quantify the impairment caused by a stroke. It consists 11 items, each item scored between zero and four (24). The higher the total score, the more severe the impairment caused by a stroke. NIHSS score will be will be used as a descriptive parameter.
• The Modified Rankin Scale (mRS) is a valid and widely used functional measure for patients after a stroke (24, 25). The scale evaluates the degree of disability or dependence in activities of daily living using a rating scale from zero (no symptoms at all) to six (dead).
• The Stroke Impact Scale (SIS) is a self-reported questionnaire that evaluates disability and health-related quality of life after a stroke and is designed for repeated administration to track changes over time (26, 27). The SIS consists of eight subscales (strength, hand function, mobility, activity of daily living, emotion, memory, communication and participation) comprising a total of 59 questions. Patients can rate each item on a five-point rating scale (from 1 = could not do at all to 5 = not difficult at all). Final scores range from zero to 100. The SIS includes an extra question on how much the patient feels that she/he has recovered from stroke that is rated on a scale from zero to 100.
• The 36-Item Short Form Healthy Survey (SF-36) is a generic patient-reported outcome measure that quantifies health status and measures health-related quality of life (28, 29). It consists of eight subscales (physical functioning, role limitations due to physical problems, general health perceptions, vitality, social functioning, role limitation due to emotional problems, general mental health, and health transition) with eight scales scores, which are the weighted sums of the questions in their subscale (zero to 100). The lower the score the higher the impairment.
• The EuroQoL EQ-5D-5l is a patient-reported outcome measure regarding the patients' health-related quality of life (30). It consists of six questions about an individuals' quality of life in general, a visual analogue scale with a score from zero to 100 and descriptive questions about five dimensions: mobility, self-care, activity of daily living, pain and complaints, fear and depression. Each dimension can be evaluated using a five-point rating scale (from 1 = no problems/pain/fear at all to 5 = extreme problems/pain/fear). The descriptive system of the EQ-5D-5l produces a 5-digit health state profile, whereas the value sets are anchored on 11,111 representing complete health.
• The German Life-Space Assessment (LSA-G) evaluates the patients' life space and range of mobility. The LSA-G consists of a questionnaire on five different life spaces: rooms besides the bedroom, area outside the house, neighborhood, town or city lived in, outside of the town or city lived in. For each life space individuals are asked whether they visited these spaces and how often, whether they needed assistive devices or personal assistance (31).
• The patient's activity level will be assessed using the GENEActiv (Activinsights, Kimbolton, UK) activity tracker. The activity tracker is a lightweight and waterproof raw data accelerometer, designed for health care research and clinical trials. It allows to monitor physical activity, sleep and everyday living through measuring acceleration, posture changes, sleep/wake time, movement activity and intensity. Patients will wear the GENEActiv sensor during inpatient rehabilitation and during the last three weeks of the intervention period. Patients' time spent in different activity levels, steps per day, and energy expenditure will be determined.
• An additional measure to evaluate the patients' physical performance is the Short Physical Performance Battery (SPPB) that combines the results of gait speed, chair raise and balance tests (32). The SPPB can be used as a predictive tool for possible impairments and can aid in the monitoring of function in older people. The scores range from zero = worst performance to twelve = best performance.
• Blood pressure and heart rate will be measured with a digital blood pressure measurement device (Medisana BU 510, medisana GmbH, Neuss, Germany) provided by the study center. Patients are reminded to measure their blood pressure every morning using the digital blood pressure measurement device and enter their data in the mobile application. During their practice sessions with the therapist (one to three), patients practice the self-measurement. For further practice, patients will be provided a video with the instructions and they can contact their coach if they have questions.
• The widely used and well accepted Timed Up and Go (TUG) test will be used to evaluate the patients' mobility and their risk of falling. Patients are asked to stand up from a chair, walk three meters, turn around, walk back and sit down. The time patients need to complete the task will be measured in seconds (33).
• The Trunk Impairment Scale (TIS) assesses static (with three items) and dynamic (with four items, each repeated two or three times) sitting balance and trunk coordination (two items, each repeated twice) in sitting position in patients after a stroke (34). Score ranges from zero to 23, where the higher score indicates better balance and coordination.
• Furthermore, a weekly reporting scheme will document progress of the investigation, serious and non-serious adverse events and device deficits. Potential harm will be assessed by the following measures: recurrent stroke, serious adverse events (death, life threatening, in-patient hospitalization or prolongation of existing hospitalization, persistent disability).

### 2.5 Sample size and statistical analyses

The sample size was defined based on relevant scientific literature that suggests sample sizes ranging between 24 and 50 participants (35, 36) for the justification of a feasibility study. Based on this recommendation, a convenient sample size of 40 patients was determined to cover different stages of the modified Rankin Scale.

Data from all participants will be analyzed quantitatively using JASP (0.18.0, The Jasp Team, Amsterdam, The Netherlands), R Studio (2023.06.0, Posit PBC, Boston, MA, USA) and Microsoft Excel (Excel 2019, Microsoft Corporation, Redmond, WA, USA). No sub-group analysis or interim analysis are planned.

**Primary analyses:** Quantitative data analysis will include descriptive statistics of all primary outcome variables: process-related parameters, training-related parameters and mHealth-related parameters. Raw count (absolute and relative frequencies (n,%) will be presented for count and nominal data, medians (range, 25th and 75th percentiles) will be reported for ordinal data and means (+/- standard deviation) will be reported for continuous data.

The START program on the Blended Clinic platform will be considered feasible and useful if at least half of the following criteria are met:

• Sample size: ≥75% of targeted sample size will be achieved
• Recruiting rate: ≥40% of informed patients will be enrolled
• Drop-out rate: ≥80% of enrolled patients will complete study participation
• Adherence: ≥70% of planned trainings will be completed
• Stability and maintenance: ≤3 system failures per week
• Safety: number of (serious) adverse events in <10% of all participants
• SUS: scores above 70 represent an acceptable usability, with scores smaller than 50 being judged as unacceptable (16)
• G-MAUQ: average scoring ≥4
• AQUA: average scoring in each dimension ≥3
• NPS: average satisfaction ≥50%
• Satisfaction (VAS): average satisfaction ≥50%
• Perceived change (VAS): health status at least remained the same

Secondary analysis will include descriptive statistics of all secondary outcome variables and pre-post comparisons of clinical and performance-based parameters. Raw count (absolute and relative frequencies (*n*, %) will be presented for count and nominal data, medians (range, 25th and 75th percentiles) will be reported for ordinal data and mean (including standard deviation) will be reported for continuous data. Regarding pre-post comparisons, Wilcoxon signed-rank test will be used for ordinal data and *t*-test for continuous data after testing for normal distribution graphically using QQ-Plot and analyzed using the Shapiro-Wilk test. Significance will be determined with *p*-value of ≤0.05.

### 2.6 Quality assurance and control

#### 2.6.1 Data handling and record keeping/archiving

All essential clinical investigation related documents will be stored securely in the research department of the Reha Rheinfelden. Participant information will be stored in locked file cabinets in areas with limited access. All reports, data collection, process and administrative forms will be identified by a coded ID number only to maintain patient confidentiality. All records that contain names or other personal identifiers, such as locator forms and informed consent forms, will be stored separately from study records identified by code number. All local databases will be secured with password-protected access systems. Forms, appointment books, and any other listings that link participant ID numbers to other identifying information will be stored in a separate, locked file in an area with limited access.

#### 2.6.2 Early termination criteria, discontinuation or modifications of the intervention

The Sponsor may terminate the investigation prematurely according to certain circumstances: ethical concerns, insufficient patient recruitment, when the safety of the patient is doubtful or at risk, alterations in accepted clinical practice that make the continuation of the investigation unwise, early evidence of benefit or harm of the experimental intervention.

The study may be stopped for safety reasons, e.g., patients rate exercise difficulty, exhaustion and pain too high after each exercise of their training program. The individual coach monitors ratings and can adapt the training program at any time according to the patient's needs. For example, this includes changing the type, intensity of the exercise and/or number of repetitions or exercises. No modifications on training frequency or on the medical device are planned.

#### 2.6.3 Study monitoring, audits and inspections

The source data and documents will be made accessible to monitors and questions are answered during the monitoring by the Sponsor-Investigator and the site staff. Monitoring will be organized as co-monitoring according to the monitoring plan and will conducted internally by members of the research group, who are not related to the research project. Four monitoring visits will be conducted: a site initiation visit, two routine monitoring visits (after enrolment of five and 25 patients) and a closeout visit after trial completion. The external monitoring will take place after enrolment of the fifth patient.

During each monitoring visit, the following documents will be checked: contracts, patient enrolment logs, informed consent forms and case report forms. File structures and folder contents will be controlled and source data verification performed.

No additional auditing is intended for the study. Study documentation and the source data/documents are accessible to auditors and inspectors from local authorities, e.g., from the ethics committee. Questions are answered during inspections. All involved parties must keep the patient data strictly confidential.

#### 2.6.4 Data management

All coded patient data as well as results from assessments and questionnaires are recorded in electronic Case Report Forms (eCRFs) for each patient by the study personnel at the Reha Rheinfelden directly during the visits using REDCap [Version 14.2.1, (Vanderbilt University, Nashville, TN, USA)]. Data recorded through the mobile application is stored on a centralized Blended Clinic AI Server in Switzerland. These data will be directly exported from the server (stored in the clinic's server) and transferred to the eCRF. Only study personnel has access to these source data. Verification of electronic captured data will take place on a regular basis, after the enrolment of five and 25 participants as described in the study monitoring plan. Electronic data is verified by crosschecking of entered data by other members of the research group and by the monitor.

#### 2.6.5 Confidentiality and data protection

Direct access to source documents will be permitted for purposes of monitoring, audits, and inspections. For external audits, monitoring events, and inspections authorized personnel (CEC, CA) will have direct access to all study data. Patient data will be treated as strictly confidential and all documents will remain in the secured and locked office of the trial center. After study completion, all documents (paper and electronic version) will be stored securely for 10 years after the end of the project, i.e., password-protected and with limited access.

## 3 Discussion

The aim of the study is to evaluate the Swiss tele-assisted rehabilitation and training program on the mobile application platform Blended Clinic AI for its feasibility, safety and performance in patients in the subacute phase after stroke. Tele-assisted rehabilitation and training programs offer a valuable opportunity to enhance recovery after stroke (37). The physical training program delivered via a mobile application has already been successfully integrated into the rehabilitation program for patients in the chronic phase of stroke recovery within outpatient settings (13). START program itself aims to facilitate the patients' transition from an inpatient to an outpatient setting.

### 3.1 Strengths

The longitudinal study design allows us to monitor the patients over a period covering two weeks during inpatient stay and 12 weeks during outpatient period. It is planned to include 40 patients. With this large number of patients, we will be able to evaluate the application in patients with a wide range of motor and/or cognitive impairments following stroke covering several stages of the mRS (24). It will be possible to make targeted adjustments in the START program for a specific level of impairment.

A key strength of the study is that it builds upon a robust foundation established by a prior usability analysis involving primary (patients) and secondary (therapists) users of the mobile application. The findings from this previous evaluation have informed important refinements and adjustments to the current study, ensuring that the START program on the Blended Clinic platform is more user-friendly and better aligned with patient needs. This iterative process of improving the study design based on direct feedback is a clear advantage, as it enhances the feasibility and practicality of the intervention. Moreover, the current study's results will play a pivotal role in planning a future randomized controlled trial, which will assess the efficacy of the intervention.

In our current study, the primary focus lays on the feasibility, safety, and performance evaluation and is designed in accordance with ISO 14155, the international standard for clinical investigations of medical devices involving human participants (38). Compliance with ISO 14155 is essential for studies involving mobile health applications, as it not only guarantees that the research is conducted in a controlled and reliable manner but also facilitates the eventual certification process and increases the likelihood that the START program on the Blended Clinic platform can be effectively integrated into clinical practice.

Lastly, another strength is the collection performance-based and mobility information combined with real-time physiological measurements, such as blood pressure monitoring. The combination provides valuable and comprehensive data on the exercise intensity, program dosage, and patients' health status changes from an inpatient to an outpatient setting.

### 3.2 Limitations

We are aware of some limitations of the study. To recruit the intended sample size within a limited time of six months only, is ambiguous. However, based on our previous usability study, we assume that at least one-third of our patients would be eligible to participate, which provides some reassurance that the study population will be adequate for initial analysis.

While some of our patients may be comfortable with technology, others may struggle with the use of the mobile application, the smartphone or tablet. Additionally, the use of older smartphones, which may not support current operating systems or applications, poses a significant barrier to participation. This issue is particularly relevant given that older patients are more likely to have outdated devices, making it difficult to implement the study's interventions effectively across all users.

Stroke patients are asked to self-rate their disability using the modified Rankin Scale (mRS) (25), a task that is typically performed by medical personnel. The mRS is a widely used tool for assessing the level of disability or dependence in daily activities following a stroke, but it can be complex and subjective for patients and could lead to an underestimation or overestimation of their functional status.

The patient-reported outcome measures used in the study, e.g., the SIS, may be too time-consuming for patients to complete them via the mobile application (26). For patients with cognitive or visual impairments, the extensive nature of these forms could be particularly challenging.

In addition, the font size of the text displayed within the app may not be optimal for older individuals or those with vision impairments, potentially limiting the usability of the mobile application in particular or digital tools in general. However, the individual therapist or coach can guide the patients.

A last limitation of the study focusses on the availability of the supervision and support for patients in the outpatient phase. Therapists or coaches are only available during regular weekday business hours. This restriction may not adequately address the needs of patients, who may require assistance outside of these hours, particularly during evenings or weekends when they may encounter difficulties using the mobile application or performing exercises. However, this coaching service would not be available to all other patients not enrolled in our clinical study.

### 3.3 Trial status

Patient recruitment stated in July 2024. The actual version of the protocol is version 1.2 from the 11. November 2024. So far, we screened 217 patients after a stroke, 51 patients were asked to participate and 19 patients agreed to participate. We expect to complete patient recruitment by the end of March 2025.

## Data Availability

As this is a study protocol, no data have been collected so far. Inquiries regarding the content of the publication can be directed to the corresponding author.
